# Polyhydroxyalkanoate production in *Priestia megaterium* strains from glycerol feedstock

**DOI:** 10.1371/journal.pone.0322838

**Published:** 2025-04-30

**Authors:** Andrew J. Cal, Victor J. Chan, Winston K. Luo, Charles C. Lee

**Affiliations:** 1 Olipha, Inc., Wilmington, Delaware, United States of America; 2 USDA-ARS-Western Regional Research Center, Bioproducts Research Unit, Albany, California, United States of America; UPES: University of Petroleum and Energy Studies, INDIA

## Abstract

Our society relies heavily on plastic, but most plastic is petroleum-based and non-biodegradable resulting in major negative environmental impacts. Polyhydroxyalkanoates (PHAs) are a type of biopolymer that can be produced from microorganisms cultured on renewable feedstocks, such as glycerol. PHAs are biodegradable and have properties similar to petroleum-based plastics. Several *Priestia megaterium* isolates have been demonstrated in previous studies to produce PHA when cultured on glycerol, but there has been no comparison of strains available in public repositories. Such comparison would be useful to identify the most promising strains for further development. In this study, we screened a number of *P. megaterium* strains from readily accessible repositories for their ability to produce PHA from glycerol. The strains had a wide range of growth and PHA production characteristics on glycerol: cell dry weight (0.5–5.7 g/L), percent PHA (4–42%), PHA titer (0.1–1.9 g/L), and yield (26–303 mg/g). The time course of PHA production varied widely among the different strains. There was also a dramatic difference in molecular weights which ranged from 119 kD to 402 kD. This information will be valuable to groups in selecting a PHA strain to develop based on their specific requirements.

## Introduction

Modern society is dependent on the benefits of petroleum-based plastics in all aspects of our economy; however, there are significant negative environmental consequences of this lifestyle. Traditional plastic production results in the release of harmful greenhouse gases into the atmosphere, and the plastic itself is generally not biodegradable thus polluting the land and water for many years. Therefore, there are intense efforts to develop sustainable alternatives. One promising approach is the use of polyhydroxyalkanoates (PHAs) which are biodegradable polyesters produced by certain microorganisms [1,2]. The characteristics of PHAs vary widely based on their specific subunit composition, and these polymers can emulate the properties of many different traditional plastics [3,4]. The most commonly produced PHA is poly-3-hydroxybutyrate (PHB) which is composed of 3-hydroxybutyrate subunits. PHB has properties (tensile strength [~30 MPa] and modulus [~3.25 GPa]) that are similar to polypropylene [5].

A major barrier to broad adoption of PHA polymer is the higher cost compared to traditional plastics. One of the major expenses of PHA production is the feedstock used to culture the microorganisms. To alleviate this problem, waste streams from different processes have been explored as feedstocks for PHA production [6–10]. Recently, glycerol has emerged as a promising substrate because it is a major byproduct of biodiesel manufacturing [11–13]. The production of biodiesel has been increasingly yearly and is predicted to reach 50 billion liters by 2030 [14,15]. Approximately, 1 g of glycerol is generated for every 10 g of biodiesel. Although there are currently applications for glycerol that do not involve PHA production, the amount of glycerol made available through the biodiesel industry far exceeds the current market.

The bacterium *Priestia megaterium* (formerly known as *Bacillus megaterium*) is an excellent candidate to produce economical levels of PHA. *P. megaterium* was the first microorganism discovered to produce PHA, specifically PHB [16]. The bacterium has been widely used in biotechnological applications to produce industrial biochemicals [17]. The fact that *P. megaterium* is a Gram-positive bacterium is an advantage over Gram-negative bacteria containing contaminating lipopolysaccharide endotoxins. There are many reagents available to genetically engineer *P. megaterium* [18,19]. *P. megaterium* is capable of growing on a wide variety of feedstocks, and economic modeling has demonstrated that it is feasible to commercially manufacture PHB from glycerol [20]. To produce PHB, *P. megaterium* uses a heteromeric PHB synthase enzyme composed of PhaC and PhaR subunits [21].

Multiple research groups have demonstrated PHA production from *P. megaterium* using glycerol as a feedstock; however, the majority of these studies utilize isolates not available in public strain repositories [20,22–30]. Additionally, experimental culturing conditions vary greatly between these studies, making direct comparisons impossible.

In order to facilitate further development of *P. megaterium* for PHA production and select appropriate strains for functional modification by genetic engineering [31], we have conducted a side-by-side comparison of 12 publicly-available strains in this study. *P. megaterium* strains were compared for their ability to produce PHB from glycerol feedstock under identical experimental conditions. Cell biomass, polymer percent, titer, and yield were monitored over the course of time. Polymers were analyzed for monomer composition, molecular weight, and thermal properties. Knowledge of the PHB production characteristics will allow groups to make informed decisions about which strain to utilize and further develop based on their industrial priorities. All the strains in this study are easily obtainable from cell repositories available to the public.

## Materials and methods

### Strains and media

Twelve *P. megaterium* strains were acquired from readily available public sources: ATCC (American Type Culture Collection; Virginia, USA), BGSC (Bacillus Genetic Stock Center, Ohio, USA), Boca Scientific Inc. (Massachusetts, USA), DSMZ (German Collection of Microorganisms and Cell Cultures, Braunschweig, Germany), and NRRL (ARS Culture Collection, Illinois, USA). All chemicals and reagents were obtained from Sigma-Aldrich (MO, USA) unless otherwise specified. Growth media was adapted from Cardozo et al. [31] and was composed of the following (per L): 1.0 g yeast extract, 1.0–3.0 g (NH_4_)_2_SO_4_, 0.2 g MgSO_4_·7H_2_O, 20.0 g carbon substrate (glycerol or glucose), 1.5 g KH_2_PO_4_, and 3.6 g Na_2_HPO_4_, and 1.0 mL of trace element solution. The trace element solution was adapted from Kim et al. [32] and was composed of the following (per L): 10.0 g FeSO_4_·7H2O, 2.25 g ZnSO_4_·7H2O, 1.0 g CuSO_4_·5H_2_O, 0.364 g MnSO_4_·H_2_O, 2.0 g CaCl_2_·2H_2_O, 0.121 g Na_2_B_4_O_7_, 0.11 g (NH_4_)_6_Mo_7_O_24_·4H_2_O, and 10.0 mL 35% HCl. For solid media, recipe was supplemented with 3 g/L NH_4_SO_4_ and 1.5% agar.

### 16S rRNA sequencing

Cultures were grown in media supplemented with 3 g/L NH_4_SO_4_. Genomic DNA was isolated from the cells using PureLink Genomic DNA Minikit (ThermoFisher Scientific, MA, USA). Genomic DNA was used as template in a PCR reaction using Herculase II Fusion DNA polymerase as described by the manufacturer (Agilent, CA, USA). The PCR primers used to amplify the 16S rRNA gene are listed below [33]:

27f: 5’- agagtttgatcmtggctcag-3’

1525r: 5’- aaggaggtgwtccarcc-3’

The resulting PCR product was processed with the DNA Clean & Concentrator-5 kit (Zymo Research, CA, USA) and sequenced with the 27f and 1525r primers. DNA Sanger sequencing was conducted by Elim Biopharmaceuticals (CA, USA). All sequences were deposited into the NCBI database (NIH, MD, USA).

### PHB production

Liquid cultures were shaken at 225 rpm at 30°C. Starter cultures were grown by inoculating 50 ml media (supplemented with 3 g/L NH_4_SO_4_) in 250 ml flasks using a colony picked from a freshly streaked agar plate. After overnight growth, the starter cultures were used to inoculate 200 ml media (supplemented with 1 g/L NH_4_SO_4_) in 1 L baffled flasks to OD_600_ = 0.2. Cultures were grown for 96 h, and time points were collected periodically. Cultures were spun down, and cell pellets were washed with 1% NaCl. This wash procedure was repeated, and the cell pellets were frozen or lyophilized. All strains were cultured in triplicate.

### Polymer analysis: gas chromatography and mass spectrometry

To 10 mg of lyophilized bacterial sample, 1 ml of methanolysis reagent (15% sulfuric acid and 85% methanol) and 1 ml chloroform were added to a 12-ml glass tube and heated at 105°C for 2 hours. After cooling on ice, 1.5 ml water was added, and the mixture was vortexed then centrifuged 10 min at 500 x g. The chloroform layer was collected and dehydrated with 50 mg anhydrous magnesium sulfate. The samples were diluted into chloroform and analyzed as previously described [34]. In brief, 2 μl were injected onto ThermoScientific Trace 1310 gas chromatograph) equipped with a TG-5MS column (ThermoFisher Scientific). Products were analyzed using a coupled ThermoScientific IQS LT Single Quadrupole Mass Spectrometer. Quantification and peak identification were obtained using the ThermoScientific Chromeleon software. Besides 3-hydroxybutyrate, no signals for other 3-hydroxy fatty acids (3-hydroxyvalerate/3-hydroxyhexanoate) were detected. A standard curve was made with pure PHB (TianAn, Zhejiang, China) ranging from 1 to 10 mg of polymer.

### Polymer analysis: gel permeation chromatography (GPC), Fourier transform infrared spectroscopy (FT-IR), differential scanning calorimetry (DSC), and thermal gravimetric analysis (TGA)

Wet cell pellets were lysed by resuspension in lysozyme solution (27.5 mg/ml lysozyme in 50 mM potassium phosphate and 16.7 mM magnesium chloride). The suspension was incubated at 37°C for 30 min, sonicated, and centrifuged at 6,000 X g for 20 min. The PHB pellet was collected and lyophilized. Aliquots containing approximately 6 mg of PHB were solubilized in 1.8 ml chloroform at 100°C for 1 h. The solution was cooled, and 1.8 ml water was added. The solution was vortexed and centrifuged at 500 X g for 10 min. The chloroform fraction was collected and twice the volume of heptane was added to precipitate the PHB. The solution was centrifuged at 16,000 X g for 15 min, and the pellet was collected and dried.

For GPC analysis to calculate molecular weight, polymers were resuspended in chloroform to a final concentration of 1 mg/ml and injected onto an Agilent 1260 Infinity II HPLC equipped with a PLgel 10 µm MiniMIX-B column (Agilent, CA, USA). A calibration curve was developed with EasiVial GPC/SEC polystyrene size standards (Agilent) [35].

For FTIR analysis, polymers were loaded on a Nicolet iS10 instrument equipped with a diamond ATR. For each sample, 16 scans were averaged from 650 to 4000 cm^−1^ at a resolution of 4 cm^−1^. Spectra were normalized using OMNIC Spectra software (ThermoFisher Scientific).

For DSC analysis, polymers were characterized on a PerkinElmer DSC 8000 instrument calibrated for melting and enthalpy with indium (PerkinElmer, MA, USA). Approximately 10 mg of polymer sample was sealed in an aluminum DSC pan and subjected to a basic heat scan. Samples were held at 40°C for 1 min, then heated to 200°C at a rate of 10°C min^−1^. Samples were held at 200°C for 0.5 min, cooled to -20°C at a rate of 10°C min^−1^, and finally heated to 200°C at the same rate. Percent crystallinity (X_c_) was determined according to the formula X_c_ = (ΔH_m_/ ΔH^0^_PHB_), where ΔH_m_ is the enthalpy of fusion and ΔH^0^_PHB_ is the theoretical enthalpy of melting a pure crystal of PHB (146 J/g) [36].

For TGA analysis, polymers were characterized on a Mettler Toledo TGA-DSC 3 + instrument (Mettler-Toledo, OH, USA). Approximately 10 mg of samples were loaded into alumina crucibles and heated from 25–350°C at a rate of 10°C min^−1^ with a nitrogen flow of 60 mL min^-1^.

### Glycerol and ammonia analysis

Culture supernatant samples (200 μl) were applied to a 96-well filter plate (FiltrEX #3510; Corning, CA, USA). The filtered media samples were analyzed for glycerol on an Agilent 1100 HPLC equipped with an Aminex HPX-87H column (Biorad, CA, USA) in 0.01 N sulfuric acid in water (pH 2.4) at a flow rate of 1 ml/min. Standard curves were made from 12.5 to 200 g/L glycerol.

Ammonium measurements were made with the API Ammonia Test kit following manufacturer’s instructions (API Aquarium Pharmaceuticals, PA, USA). Absorbance at 595 nm was read on a SpectraMax M3 plate reader (Molecular Devices, CA, USA). A standard curve was made from 0.015 to 0.1 g/L ammonium sulfate.

## Results and discussion

Twelve *Priestia megaterium* strains in our collection that were obtained from publicly accessible repositories, including two common industrial strains [37], QM B1551 and DSM319, were studied (Table 1). The majority of the strains were acquired from the ARS Culture Collection (IL, USA) and were all deposited by different researchers. The strains were assayed for growth and PHB production on defined media containing glycerol as carbon substrate, and timepoints were collected for up to 96 hours (S1 Fig). Interestingly, although QM B1551 grew on solid media with glycerol substrate, this strain did not have significant growth in liquid culture. PV 586 is a plasmidless derivative of QM B1551 and likewise did not grow in liquid culture.

**Table 1:** P*. megaterium* strains examined in this study.

Strain	Source	16S rRNA NCBI accession	Alternate nomenclature
NRS 269	ATCC	OR561997*	
QM B1551	BGSC	NC_014019	
PV 586	BGSC		
YYBm1	BocaScientific		
DSM319	DSMZ	CP001982	
NRRL B-349	NRRL	OR561998*	ATCC 8245; NRS 245
NRRL B-350	NRRL	OR561999*	ATCC 7056; NRS 239
NRRL B-352	NRRL	OR562000*	ATCC 7703; NRS 615
NRRL B-1367	NRRL	OR562001*	APF 12; NRRL B-940
NRRL B-1851	NRRL	OR562002*	ATCC 11478
NRRL B-3254	NRRL	OR562003*	ATCC 11561
NRRL B-14308	NRRL	NZ_CP009920	ATCC 14581

*This study. 16S rRNA were sequenced as described in Materials and methods.

**Fig 1:**
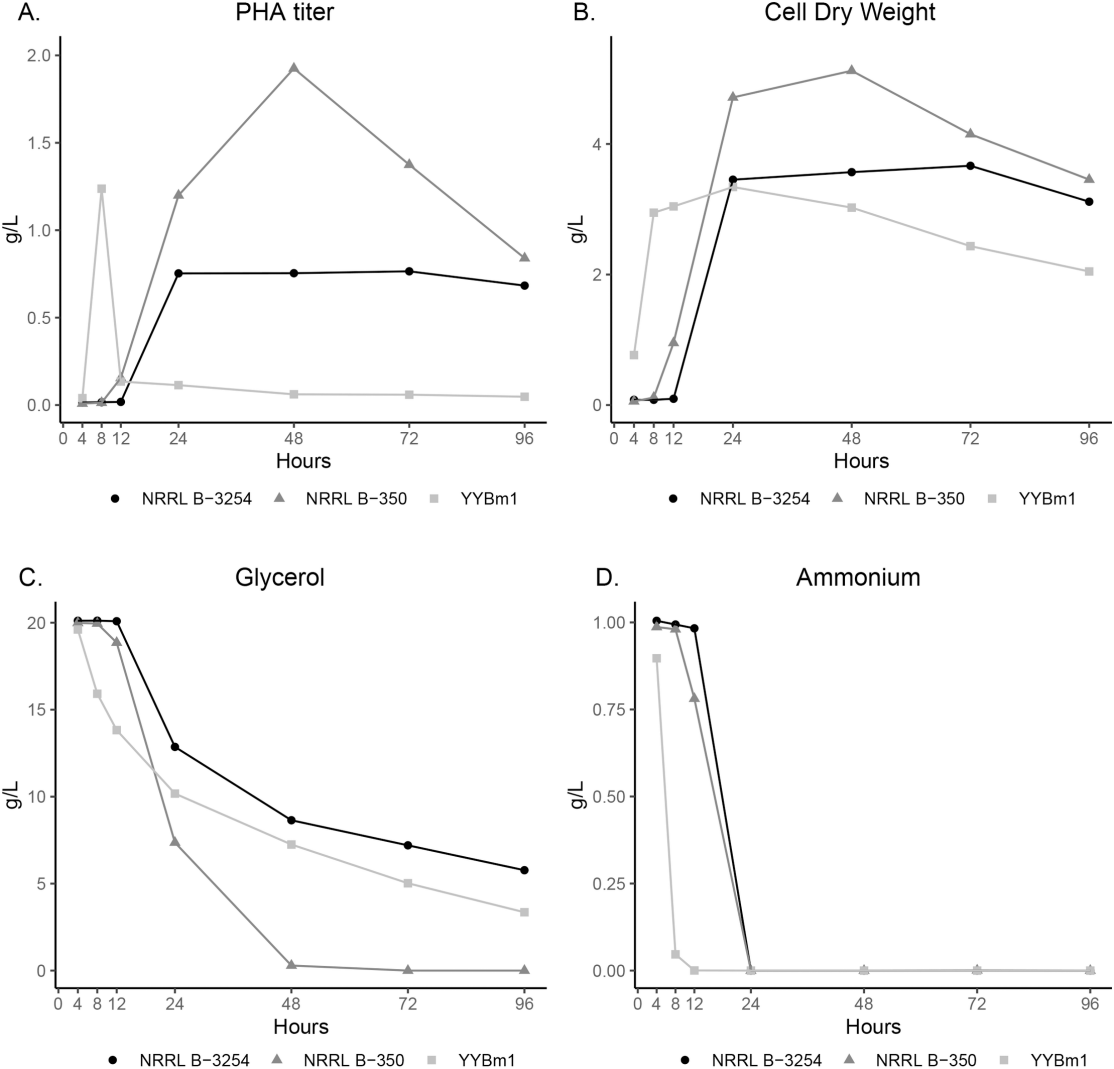
Time course of growth and PHB production of three *P. megaterium* strains. (A) PHB titer, (B) cell dry weight, (C) residual glycerol, and (D) residual ammonium. All data is the average of three replicates.

The ten *P. megaterium* strains that did grow in liquid culture demonstrated a wide variety of growth and PHB production profiles (Table 2 and S1 Fig). To illustrate these differences, three strains with distinct PHB production profiles were compared (Fig 1). YYBm1 has the fastest time of maximum PHB titer at 8 h whereas NRRL B-3254 and NRRL B-350 do not accumulate significant levels of polymer until 24 h (Fig 1A). At its peak PHB titer at 48 h, NRRL B-350 produced more PHB that YYBm1 (1.9 g/L vs 1.2 g/L). NRRL B-3254 had the lowest PHB titer of the three strains at 0.8 g/L.

**Table 2:** Growth and PHB production.

Strain	Time (max)	PHB (g/L)	CDW (g/L)	PHB (%)	Yield (mg/g)	Productivity (g/L/h)
NRS 269	24	1.8 ± 0.4	5.3 ± 1.3	34.7 ± 2.7	116 ± 10	0.08 ± 0.02
YYBm1	8	1.2 ± 0.1	2.9 ± 0.2	42.0 ± 0.5	303 ± 15	0.15 ± 0.01
DSM 319	8	0.7 ± 0.0	2.7 ± 0.2	27.5 ± 1.4	184 ± 15	0.09 ± 0.00
NRRL B-349	12	0.5 ± 0.0	2.9 ± 0.1	18.9 ± 0.8	96 ± 5	0.05 ± 0.00
NRRL B-350	48	1.9 ± 0.1	5.1 ± 0.2	37.7 ± 3.0	98 ± 6	0.04 ± 0.00
NRRL B-352	48	1.6 ± 0.2	4.4 ± 0.1	37.4 ± 4.5	83 ± 9	0.03 ± 0.00
NRRL B-1367	48	1.8 ± 0.5	4.6 ± 1.6	40.1 ± 3.1	99 ± 21	0.04 ± 0.01
NRRL B-1851	24	1.9 ± 0.1	5.7 ± 0.1	32.5 ± 2.3	114 ± 8	0.08 ± 0.00
NRRL B-3254	24	0.8 ± 0.0	3.5 ± 0.1	21.8 ± 1.0	106 ± 6	0.03 ± 0.00
NRRL B-14308	12	0.1 ± 0.0	0.5 ± 0.0	22.9 ± 1.4	64 ± 4	0.01 ± 0.0

NRRL B-3254 had the longest period of stable PHB titer with little change from 24 h to 96 h (Fig 1A). On the other hand, after the NRRL B-350 PHB titer peaks at 48 h, the amount drops steadily from 1.9 g/L to 0.8 g/L at 96 h. YYBm1 showed the greatest change in PHB titer where the titer rapidly dropped to very low levels from 8 h to 12 h.

The biomass levels of NRRL B-3254 and NRRL B-350 trend proportionally with their PHB titers with NRRL B-350 biomass decreasing after 48 h and the NRRL B-3254 biomass not showing a dramatic decrease from 24 h to 96 h (Fig 1B). On the other hand, the biomass of YYBm1 does not parallel the PHB titer. Although the PHB titer drops almost completely after 8 h, the YYBm1 biomass increases up to 24 h before gradually decreasing up to 96 h.

The consumption rates of glycerol and ammonium were initially much greater for YYBm1 compared to NRRL B-3254 and NRRL B-350 which is consistent with the more rapid initial growth rate of YYBm1 (Fig 1C and 1D). Only NRRL B-350 consumed all the available glycerol which is reflected in the highest PHB titer among the three strains at 48 h.

YYBm1 is a mutant of DSM 319 in which the *nprM* and *xylA* genes are deleted. Similar to its mutant derivative, DSM 319 also had a maximum PHB titer at 8 h (Table 2 and S1 Fig). Although the accumulated biomass (cell dry weight) of both DSM319 and YYBm1 were similar, the YYBm1 strain had a greater %PHB (42% vs 28%) and thus a higher PHB titer (1.2 g/L vs 0.7 g/L). It is unclear why the YYBm1 strain had a higher PHB titer relative to the DSM319 parent strain. It is possible that the deletion of the *nprM* and *xylA* genes allows more resources to be directed towards PHB synthesis. In addition, compared to the other *P. megaterium* strains, both the DSM 319 and YYBm1 strains had relatively narrow windows of time in which PHB was rapidly produced and then broken down after 12 h. Thus, it is possible that both strains had similar maximum PHB titers which occurred at slightly different times that were not captured in our sample collection regimen.

The sizes of the polymers were analyzed by GPC (Table 3). Among the strains that were grown on glycerol, DSM 319 had the highest molecular weight (402 kD). The other strains all had molecular weights that ranged from 119 kD to 319 kD. It is known that polymers from bacteria cultured on glycerol are typically smaller than those cultured on glucose [12]. Glycerol is known to serve as a chain terminator in PHB polymerization and can attach to the end of the polymer via the primary or secondary hydroxyl of the molecule. Previous studies have demonstrated that using glycerol as feedstock decreased the molecular weight of the polymer, and this effect was correlated with the concentration of glycerol [38,39].

**Table 3:** Gel permeation chromatography of PHB from different *P. megaterium* strains.

Strain	Mw	Mn	Mp	Mv	Mz	Mz + 1	PD
NRS 269	194,004	58,917	126,776	164,702	519,133	970,682	3.29
YYBm1	307,124	103,686	201,719	267,073	680,527	1,113,765	2.96
DSM 319	402,080	128,726	233,228	344,661	965,724	1,633,550	3.12
NRRL B-349	225,822	73,445	145,250	194,160	581,779	1,175,519	3.08
NRRL B-349 (glucose)	1,055,093	125,250	156,139	817,187	3,289,967	5,008,052	8.42
NRRL B-350	122,965	40,088	85,552	106,286	310,448	629,947	3.07
NRRL B-352	159,570	67,585	116,933	142,216	316,430	509,398	2.36
NRRL B-1367	118,774	56,722	98,298	107,886	206,790	299,807	2.09
NRRL B-1851	151,765	55,479	104,888	132,079	356,781	643,445	2.74
NRRL B-3254	318,926	74,376	128,447	257,364	1,020,734	1,756,349	4.29
NRRL B-14308	205,697	59,473	124,135	173,019	579,159	1,108,353	3.46

Although MK386891 had a higher PHB titer than NRRL B-1851, the growth time for the MK386891 was much longer (64 h vs 24 h) which translated into a higher productivity for the NRRL B-1851 (0.08 g/L/h vs 0.04 g/L/h).

All polymers were isolated from cultures grown with glycerol substrate unless otherwise specified. Mw (weight averaged MW), Mn (number averaged MW), Mp (peak MW), Mv (viscosity average MW), Mz (third moment), and PD (polydispersity). All MW are expressed in daltons.

**Fig 2:**
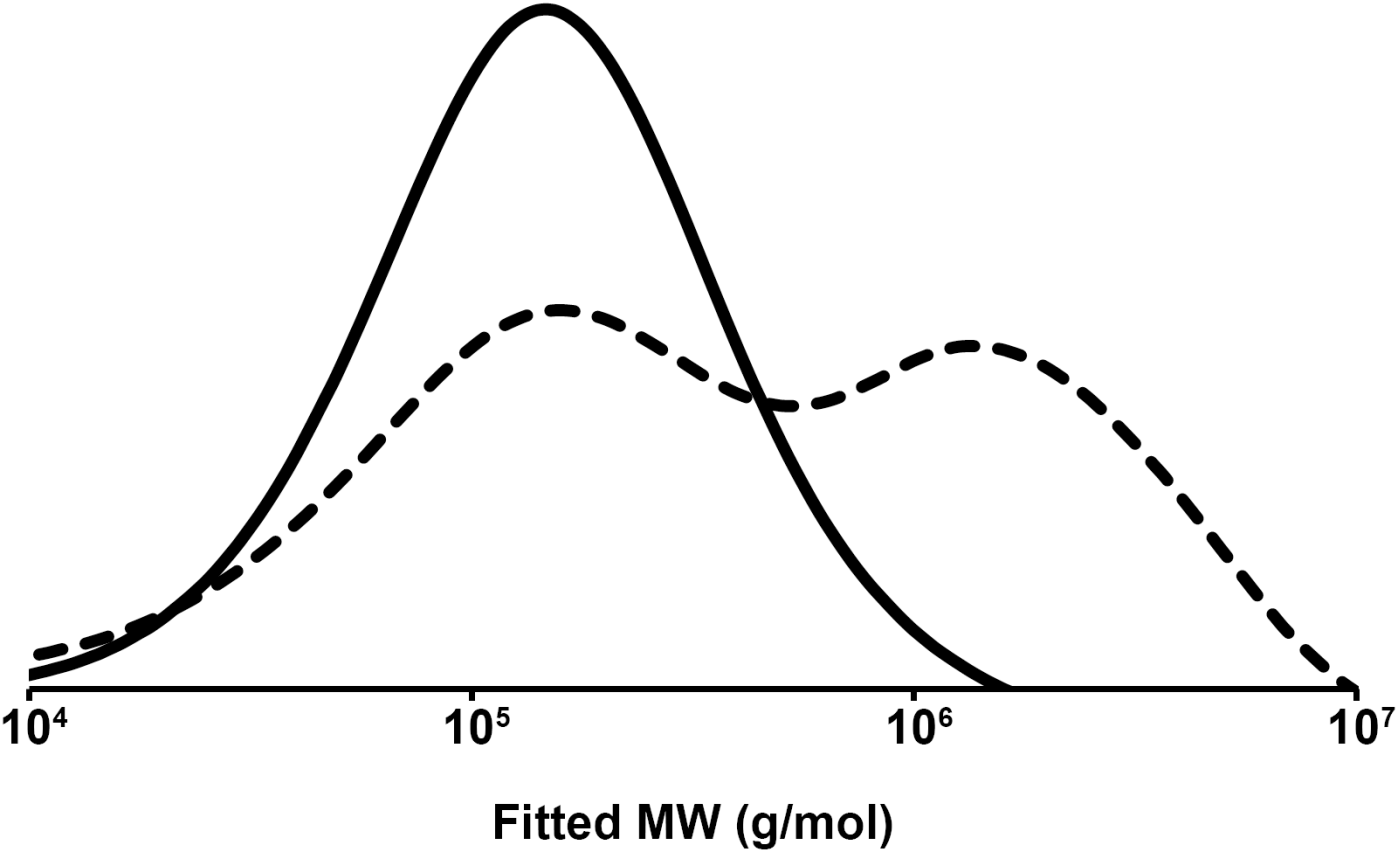
Molecular weight distributions of polymer from NRRL B-349. Polymers were extracted from cells grown in cultures supplemented with glycerol (solid) or glucose (dashed).

There is likely an additional mechanism responsible for the lower molecular weights. *P. megaterium* PHB polymerase is a Class IV synthase composed of two subunits: the main PhaC synthase and a smaller PhaR accessory [21]. The PhaRC synthase complex has been demonstrated to also have an alcoholysis scission activity [40]. The enzyme can use an alcohol to cleave the polymer internally thereby capping the smaller polymer with a new molecule [41]. When recombinant *E. coli* strains transformed with Class IV synthases were cultured on Luria Bertani (LB) media, the initial molecular weight of the polymer decreased from 10^5^ to 10^4^ Da over the course of 12 h to 60 h [42]. The GPC chromatogram of the intermediate timepoints showed the initial polymer gradually reducing in size to develop a bimodal distribution that eventually resolved to a single peak of lower molecular weight polymer. This reduction in molecular weight is believed to be caused by the alcoholysis scission activity using endogenously produced ethanol [40]. When the NRRL B-349 strain was grown in media containing glucose instead of glycerol, the intermediate time point that was analyzed showed the polymer had a higher molecular weight than that of the polymer from the culture grown in glycerol (Table 3). The GPC chromatogram of the two polymer samples showed that the polymer from the glucose culture had a bimodal distribution similar to that seen in the previous study [42] (Fig 2). It is likely that the polymer from the glucose culture would have eventually resolved into a single peak of lower molecular weight at a later time point.

**Fig 3:**
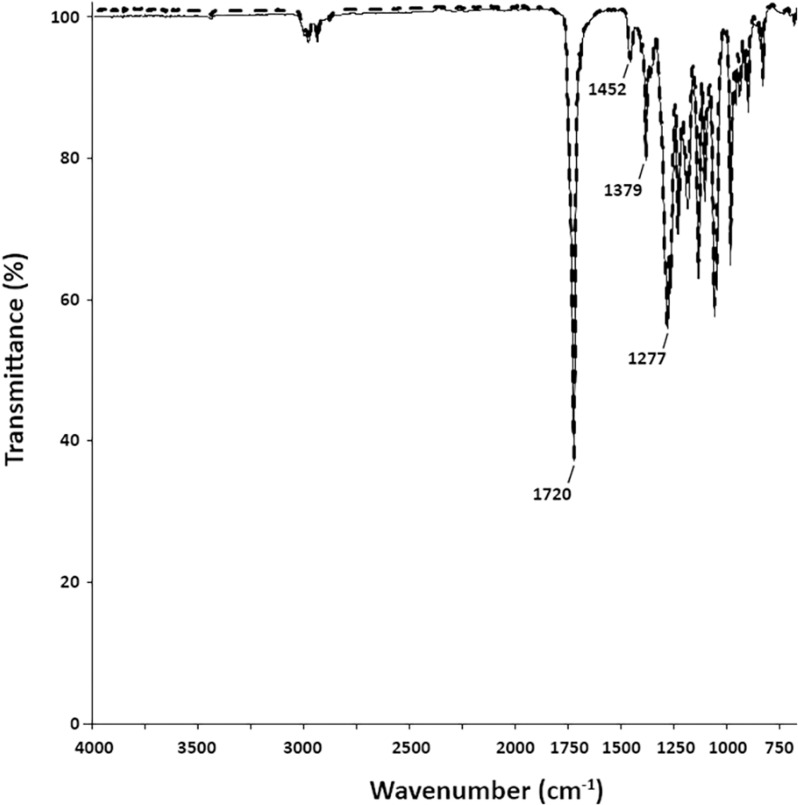
FT-IR of polymers from NRRL B-349. Polymers were extracted from cells grown in cultures supplemented with glycerol (solid) or glucose (dashed).

The polymers produced by the NRRL B-349 cultured on either glycerol or glucose were also compared by FT-IR (Fig 3). Both polymers had very similar spectra with multiple bands (1720, 1452, 1379, and 1277 cm^-1^) that are characteristic of PHB [25,31,43]. The strongest band at 1720 cm^-1^ corresponds to the ester carbonyl group. The bands at 1452 cm^-1^ and 1379 cm^-1^ are attributed to -CH_2_ and -CH_3_ groups, respectively. The thermal properties of both polymers were analyzed by differential scanning calorimetry (T_m_, T_c_, ΔH_m_, and X_c_) and thermogravimetric analysis (T_10%_ and T_max_) (Table 4). These values were similar between both polymers indicating that the thermal properties were not dramatically impacted by the difference in polymer molecular weight distribution.

**Table 4:** Thermal properties of polymers extracted from strain NRRL B-349 grown in cultures supplemented with glycerol or glucose.

	DSC.				TGA.	
**Polymer**	**T**_**m**_ **(°C)**	**T**_**c**_ **(°C)**	**ΔH**_**m**_ **(J/g)**	**X**_**c**_ **(%)**	**T**_**10%**_ **(°C)**	**T**_**max**_ **(°C)**
Glycerol-based PHB	178.4	74.2	59.5	40.8	284.2	296.0
Glucose-based PHB	180.3	73.3	64.4	44.4	281.2	293.7

T_m_, melting temperature; T_c_, crystallization temperature; ΔH_m_, enthalpy of fusion; X_c_, percent crystallinity; T_10%_ (temperature at 10% decomposition); and T_max_ (temperature at maximum decomposition rate).

There is a great deal of variation in the PHA production profile and molecular weights among the multiple strains analyzed (Tables 2 and 3) although the reason for these differences is currently unknown. The strains may have different tolerances to glycerol or metabolize the feedstock with varying efficiencies. Another possibility is that the PHB polymerases might have different activities. Thus, a more efficient PHB polymerase could lead to more and/or higher molecular weight biopolymer. The PHB polymerases might also have different alcoholysis scission activities which would also modulate the final molecular weight of the biopolymer.

Table 5 summarizes results from previous work producing polyhydroxyalkanoates from *P. megaterium* strains cultured on glycerol feedstock including strain NRRL B-1851 from this study. Many of the strains from the past studies have not been deposited in publicly accessible repositories. It is difficult to compare the different strains due to the variety of growth conditions. For instance, the culture temperatures ranged from 25°C-37°C while the timepoints analyzed varied from 12 h to 84 h. Even the glycerol in the media varied with some groups using pure lab grade glycerol while others used crude preparations of glycerol from biodiesel waste streams. In general, the highest PHB titers were derived from bioreactors employing batch and fed-batch feed strategies compared to flasks. A direct comparison was made when Moreno et al. grew *P. megaterium* strain B2 in both flask and bioreactor and obtained approximately three times more PHB in the latter [27].

**Table 5:** PHB production from *P. megaterium* cultured on glycerol feedstock.

Strain	Culture	time (h)	PHB titer (g/L)	CDW (g/L)	PHB (%)	yield (mg/g)	productivity (g/L/h)	Reference
NRRL B-1851	flask (batch)	24	1.9	5.7	33	114	0.08	This study
ASL11	flask (batch)	120	0.92	1.87	50	58	0.008	[44]
B2	flask (batch)	14	0.43	1.26*	34	NR	0.03*	[27]
BBST4	flask (batch)	48	1.14	3.8	30	38	0.02	[28]
DSMZ32	flask(batch)	72	0.66	2.68	25	20	0.009	[45]
LVN01 (BmGD)	flask (batch)	30	0.25	3.17	8	NR	0.01*	[24]
MK386891	flask (batch)	64	2.73	6.82	40	NR	0.04*	[26]
MTCC10086	flask (batch)	48	0.65	1.54	39	NR	0.01*	[23]
OU303A	flask (batch)	48	NR	NR	62	NR	NR	[30]
DSM32	flask (fed batch)	48	0.054	NR	NR	NR	0.001*	[22]
B2	bioreactor (batch)	11	1.2	3.87	31	NR	0.11	[27]
BBST4	bioreactor (batch)	42	3.4	5.7	60	NR	0.08	[25]
DSM 509	bioreactor (batch)	58	3.4	10.4	32	367	0.057	[46]
environmental isolate	bioreactor (batch)	42	4.8	7.7	62	300	0.11*	[20]
DSM 509	bioreactor (fed batch)	48	2.83	4.73	60	NR	0.06*	[29]
DSM 509	bioreactor (fed batch)	52	4.94	8.3	61	549	0.095	[46]
LVN01	bioreactor (fed batch)	36	0.72	1.91	38	NR	0.02	[24]

*Personal communication

When compared to other strains that were previously grown in flasks, several of the strains in this study had higher PHB titers (1.8–1.9 g/L) than all of the previously studied strains except for *P. megaterium* strain MK386891 (2.73 g/L).

## Conclusions

This study provides comparison of microbial PHB production using glycerol feedstock from different *P. megaterium* strains that are available from readily accessible repositories. Based on this information, strains can be selected for further production optimizations depending on user priorities. For instance, if a longer window of stable PHA titer were essential, one could select a strain such as NRRL B-350 which maintains a constant titer from 24–96 hours. Alternatively, the common industrial strain DSM 319 could be selected if production speed were most critical since PHB rapidly accumulated to the maximum titer at only 8 h. Although the PHB titer does rapidly decrease after 12 hours, the DSM 319 strain could serve as a target for genetic modification to maintain higher levels of polymer over time.

Besides the different PHB production profiles, the *P. megaterium* strains were also demonstrated to produce biopolymers in a range of molecular weights. While high molecular weight polymers are valued for their higher tensile strength, lower molecular weight PHB could also have utility if greater flexibility is required. Thus, different *P. megaterium* strains could be selected for PHB production depending on the end applications.

Regardless of the specific strain that is chosen for PHB production from glycerol feedstock, optimization of growth conditions would also be expected to increase PHB levels. Simply growing the bacteria in bioreactors should yield large improvements compared to the flask conditions used in this study. Furthermore, modifications of the culture media (temperature, pH, ammonium level, glycerol source and concentration, etc.) can all impact PHB production in *P. megaterium*.

## Supporting information

S1 Fig Growth and polyhydroxyalkanoate production time courses. (PDF)
