# Correction: Polyhydroxyalkanoate production in *Priestia megaterium* strains from glycerol feedstock

**DOI:** 10.1371/journal.pone.0327834

**Published:** 2025-07-07

**Authors:** The PLOS One Staff

The legend for Table 3 was incorrectly included in the body of the article. Additionally, a line from the Results and Discussion section was erroneously included within Table 3. Please see the correct Table 3 here.

**Table 3:** Gel permeation chromatography of PHB from different *P. megaterium* strains.

Strain	Mw	Mn	Mp	Mv	Mz	Mz + 1	PD
NRS 269	194,004	58,917	126,776	164,702	519,133	970,682	3.29
YYBm1	307,124	103,686	201,719	267,073	680,527	1,113,765	2.96
DSM 319	402,080	128,726	233,228	344,661	965,724	1,633,550	3.12
NRRL B-349	225,822	73,445	145,250	194,160	581,779	1,175,519	3.08
NRRL B-349 (glucose)	1,055,093	125,250	156,139	817,187	3,289,967	5,008,052	8.42
NRRL B-350	122,965	40,088	85,552	106,286	310,448	629,947	3.07
NRRL B-352	159,570	67,585	116,933	142,216	316,430	509,398	2.36
NRRL B-1367	118,774	56,722	98,298	107,886	206,790	299,807	2.09
NRRL B-1851	151,765	55,479	104,888	132,079	356,781	643,445	2.74
NRRL B-3254	318,926	74,376	128,447	257,364	1,020,734	1,756,349	4.29
NRRL B-14308	205,697	59,473	124,135	173,019	579,159	1,108,353	3.46

All polymers were isolated from cultures grown with glycerol substrate unless otherwise specified. Mw (weight averaged MW), Mn (number averaged MW), Mp (peak MW), Mv (viscosity average MW), Mz (third moment), and PD (polydispersity). All MW are expressed in daltons.

The last sentence of the Results and Discussion section is missing. The sentence should read as follows: Although MK386891 had a higher PHB titer than NRRL B-1851, the growth time for the MK386891 was much longer (64 h vs 24 h) which translated into a higher productivity for the NRRL B-1851 (0.08 g/L/h vs 0.04 g/L/h).

The publisher apologizes for the errors.
