# Supplementary material for: Polyhydroxyalkanoate production in Priestia megaterium strains from glycerol feedstock
Source: PLoS One. 2025 Apr 30;20(4):e0322838. doi: 10.1371/journal.pone.0322838 (PMC12043173; doi:10.1371/journal.pone.0322838)
Supplement: S1 Fig — (PDF) [file pone.0322838.s001.pdf]

Supporting Figure 1

Polyhydroxyalkanoate production in *Priestia megaterium* strains from glycerol feedstock

Andrew J. Cal[1], Victor J. Chan[2], Winston K. Luo[2], and Charles C. Lee[2]*

1. Olipha, Inc., 1209 N Orange St., Wilmington, DE 19801 USA

2. USDA-ARS-Western Regional Research Center, Bioproducts Research Unit, 800 Buchanan St., Albany, CA 94710 USA

*Charles.Lee@usda.gov

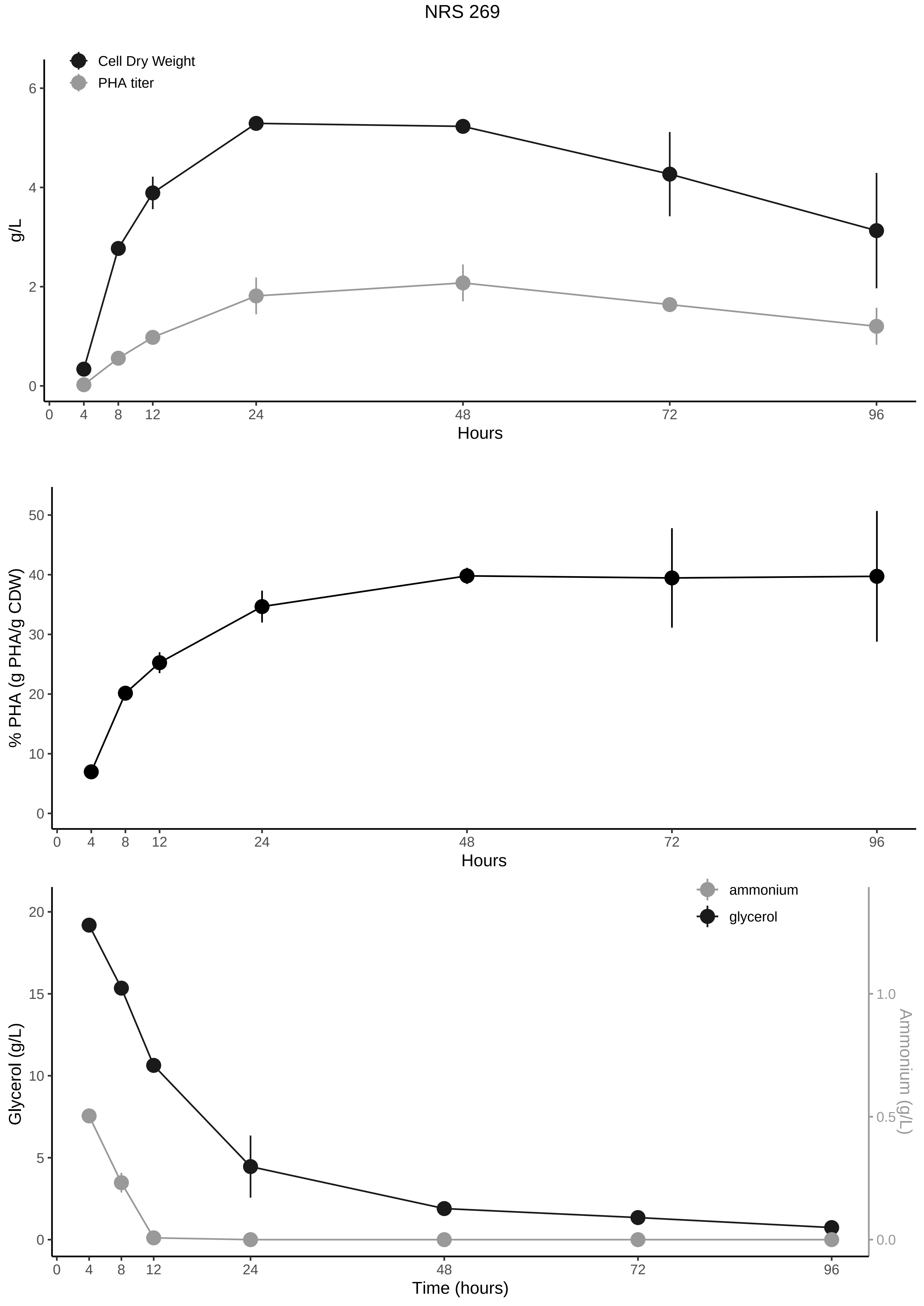
NRS 269
Cell Dry Weight
PHA titer
g/L
6
4
2
0
0
4
8
12
24
48
72
96
Hours
% PHA (g PHA/g CDW)
50
40
30
20
10
0
0
4
8
12
24
48
72
96
Hours
ammonium
glycerol
Glycerol (g/L)
20
15
10
5
0
Ammonium (g/L)
1.0
0.5
0.0
0
4
8
12
24
48
72
96
Time (hours)

YYBm1

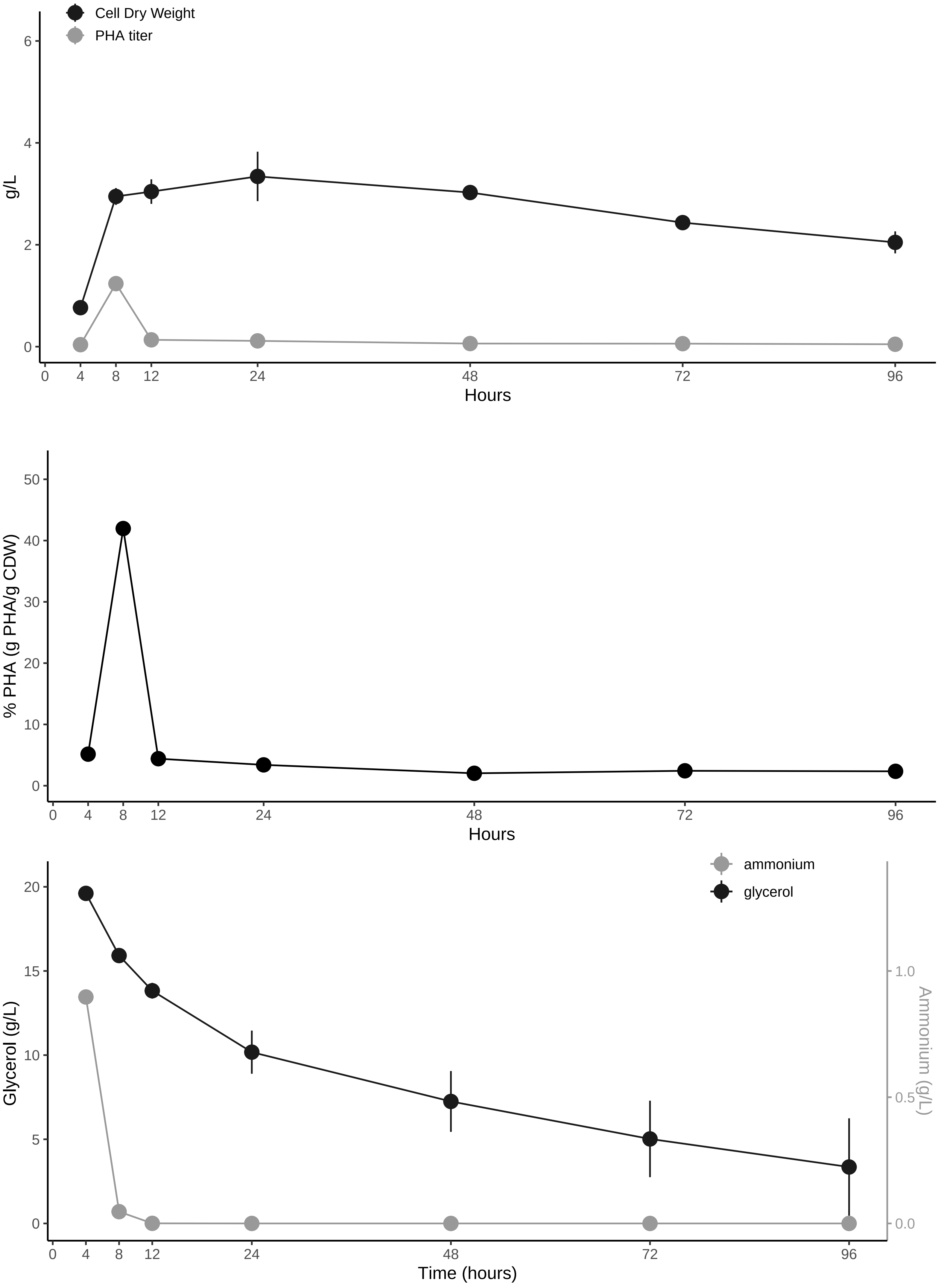

Cell Dry Weight
PHA titer
g/L
0
2
4
6
0
4
8
12
24
48
72
96
Hours
% PHA (g PHA/g CDW)
0
10
20
30
40
50
0
4
8
12
24
48
72
96
Hours
ammonium
glycerol
Glycerol (g/L)
0
5
10
15
20
Ammonium (g/L)
0.0
0.5
1.0
0
4
8
12
24
48
72
96
Time (hours)

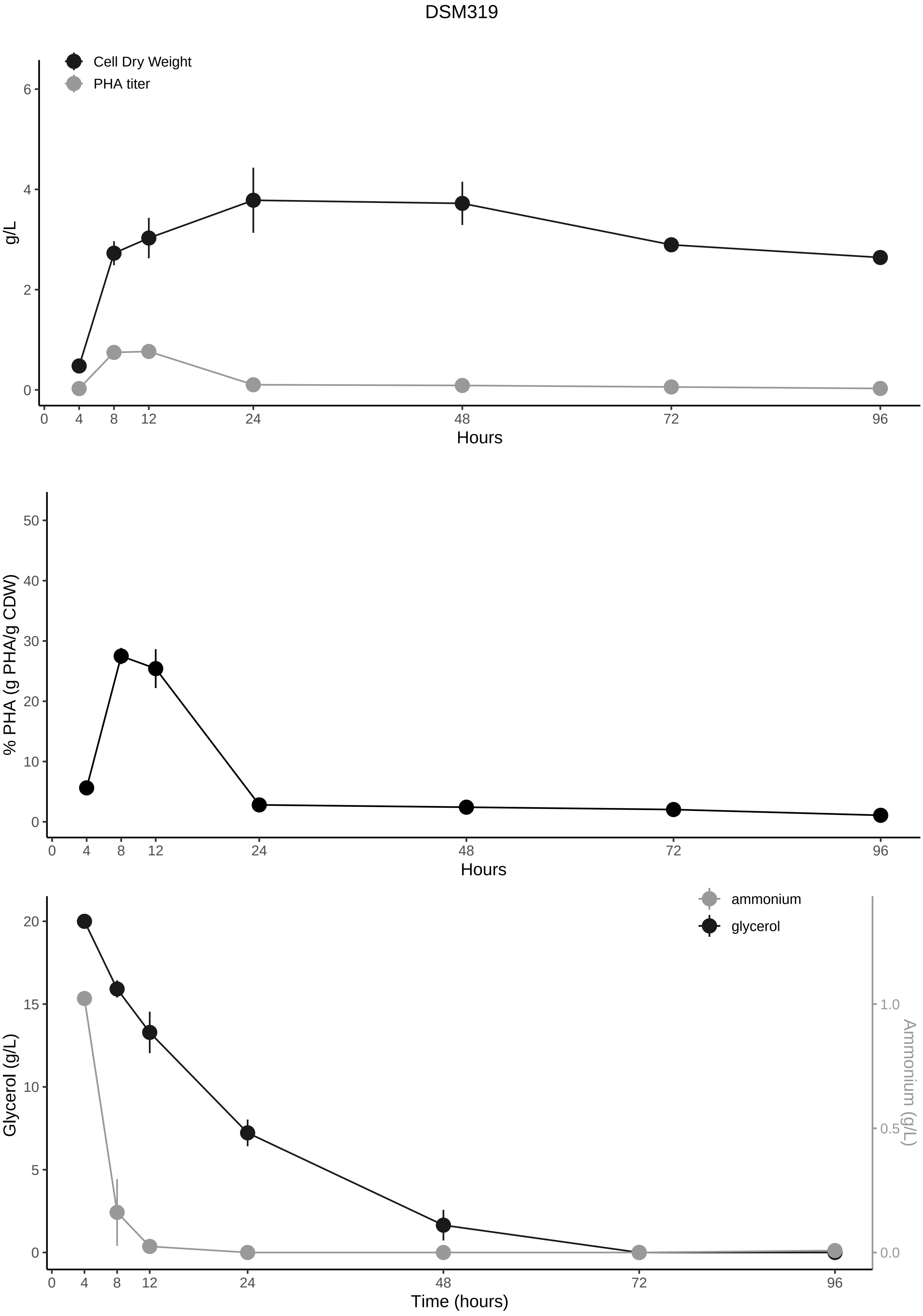

DSM319
Cell Dry Weight
PHA titer
g/L
0
2
4
6
0
4
8
12
24
48
72
96
Hours
% PHA (g PHA/g CDW)
0
10
20
30
40
50
Hours
ammonium
glycerol
Glycerol (g/L)
0
5
10
15
20
Ammonium (g/L)
0.0
0.5
1.0
Time (hours)

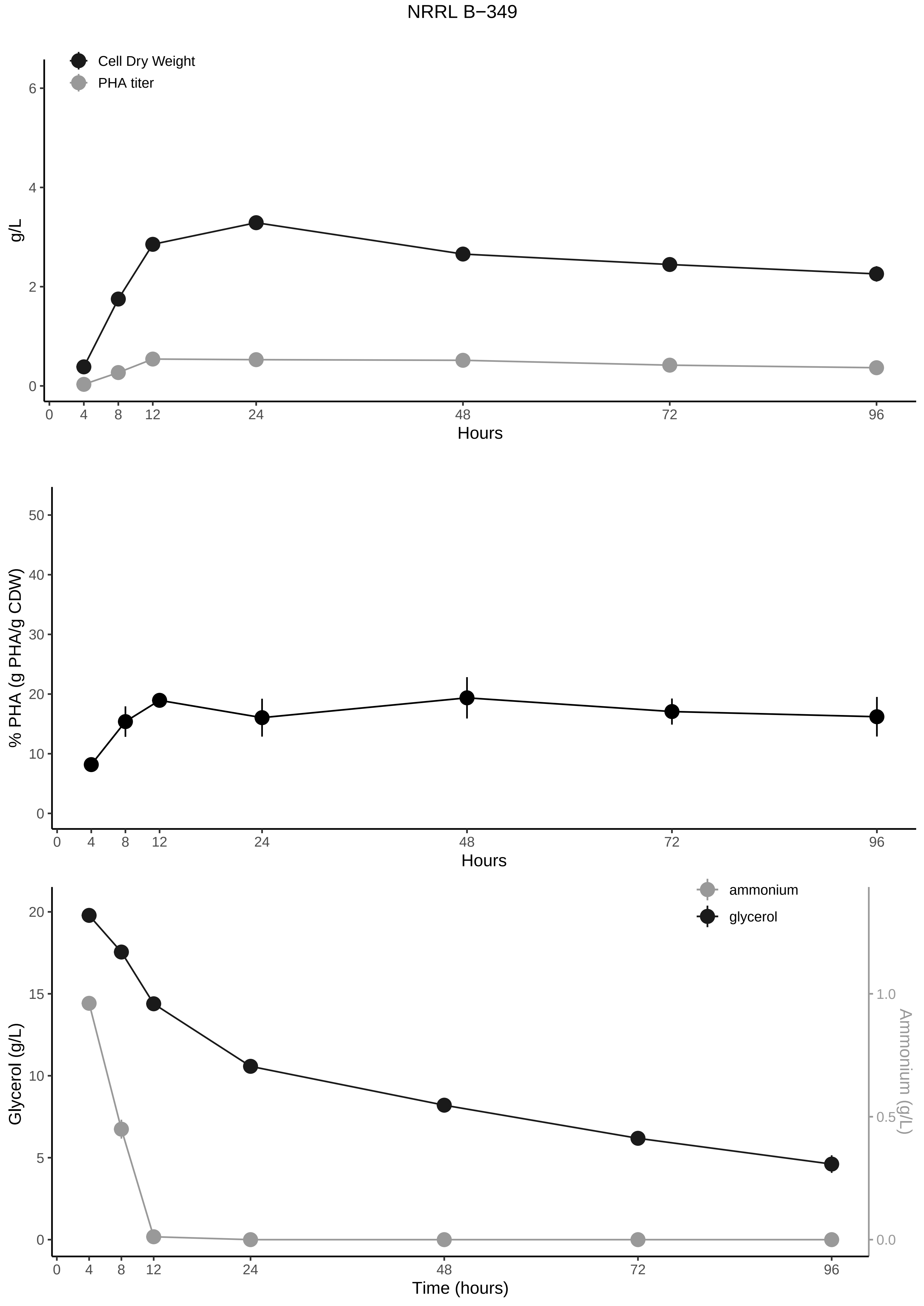

NRRL B−349
Cell Dry Weight
PHA titer
g/L
6
4
2
0
0
4
8
12
24
48
72
96
Hours
% PHA (g PHA/g CDW)
50
40
30
20
10
0
0
4
8
12
24
48
72
96
Hours
ammonium
glycerol
Glycerol (g/L)
20
15
10
5
0
Ammonium (g/L)
1.0
0.5
0.0
0
4
8
12
24
48
72
96
Time (hours)

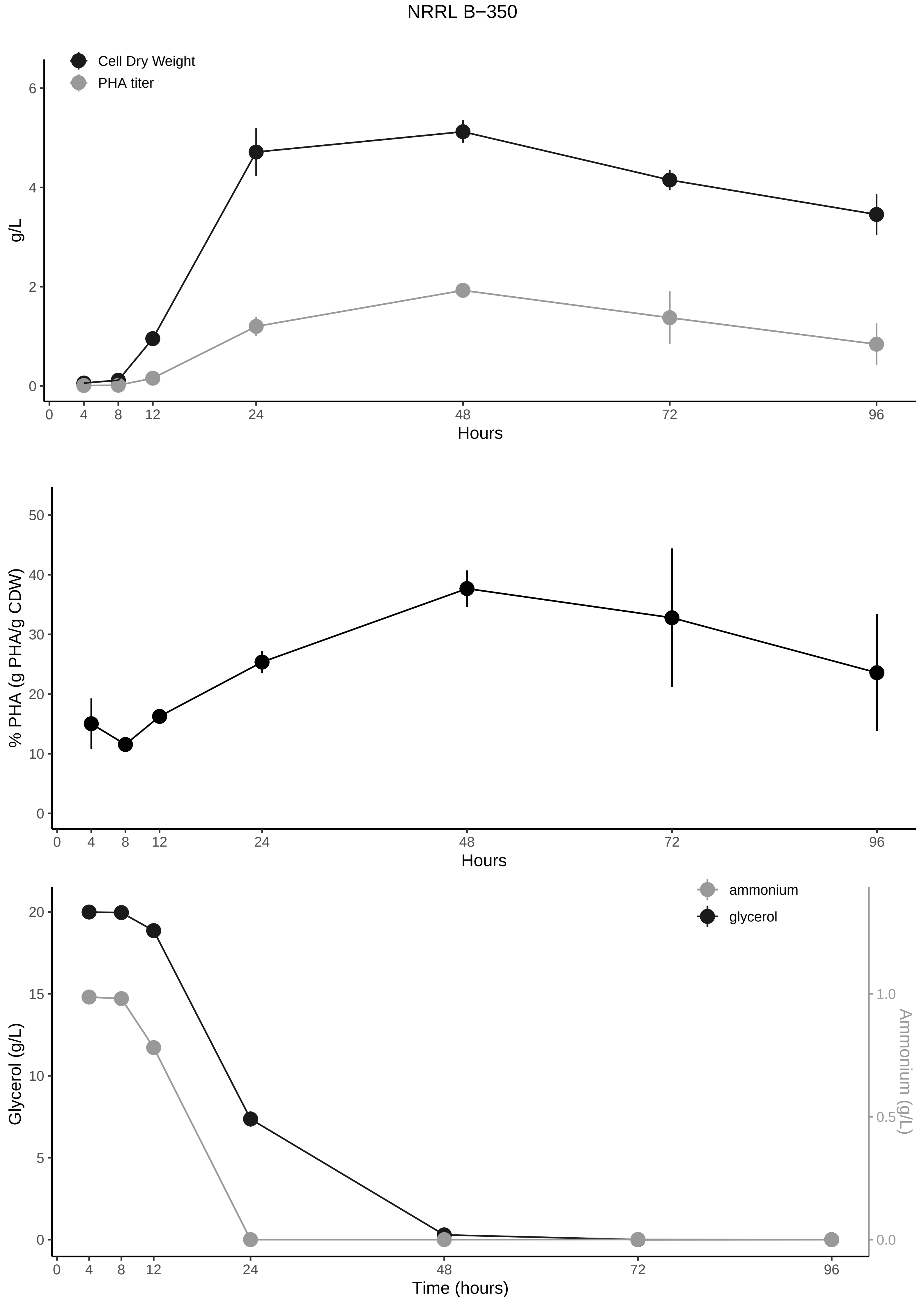

NRRL B−350
Cell Dry Weight
PHA titer
g/L
6
4
2
0
0
4
8
12
24
48
72
96
Hours
% PHA (g PHA/g CDW)
50
40
30
20
10
0
0
4
8
12
24
48
72
96
Hours
ammonium
glycerol
Glycerol (g/L)
20
15
10
5
0
Ammonium (g/L)
1.0
0.5
0.0
0
4
8
12
24
48
72
96
Time (hours)

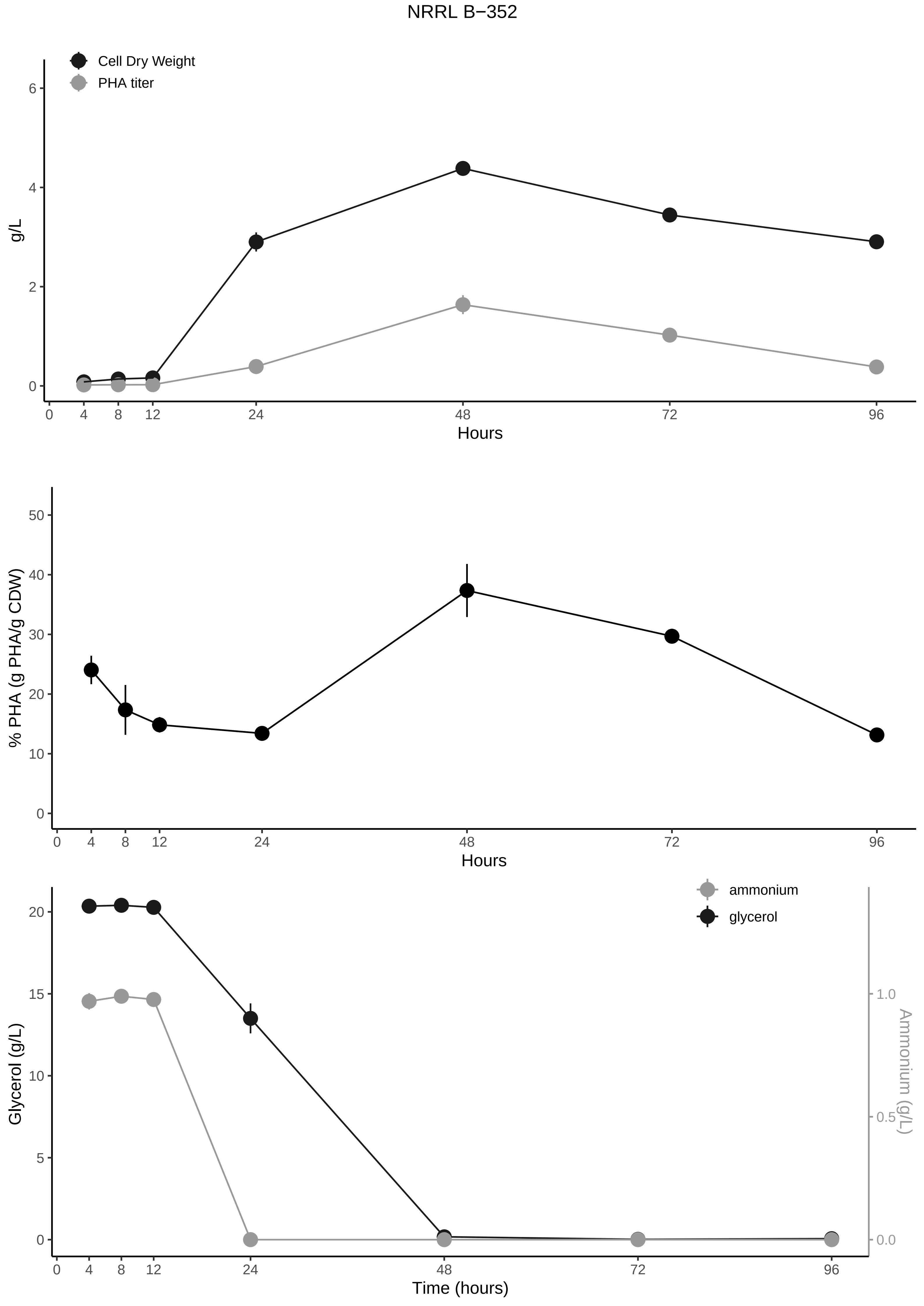

NRRL B−352
Cell Dry Weight
PHA titer
g/L
0
2
4
6
0
4
8
12
24
48
72
96
Hours
% PHA (g PHA/g CDW)
0
10
20
30
40
50
0
4
8
12
24
48
72
96
Hours
ammonium
glycerol
Glycerol (g/L)
0
5
10
15
20
Ammonium (g/L)
0.0
0.5
1.0
0
4
8
12
24
48
72
96
Time (hours)

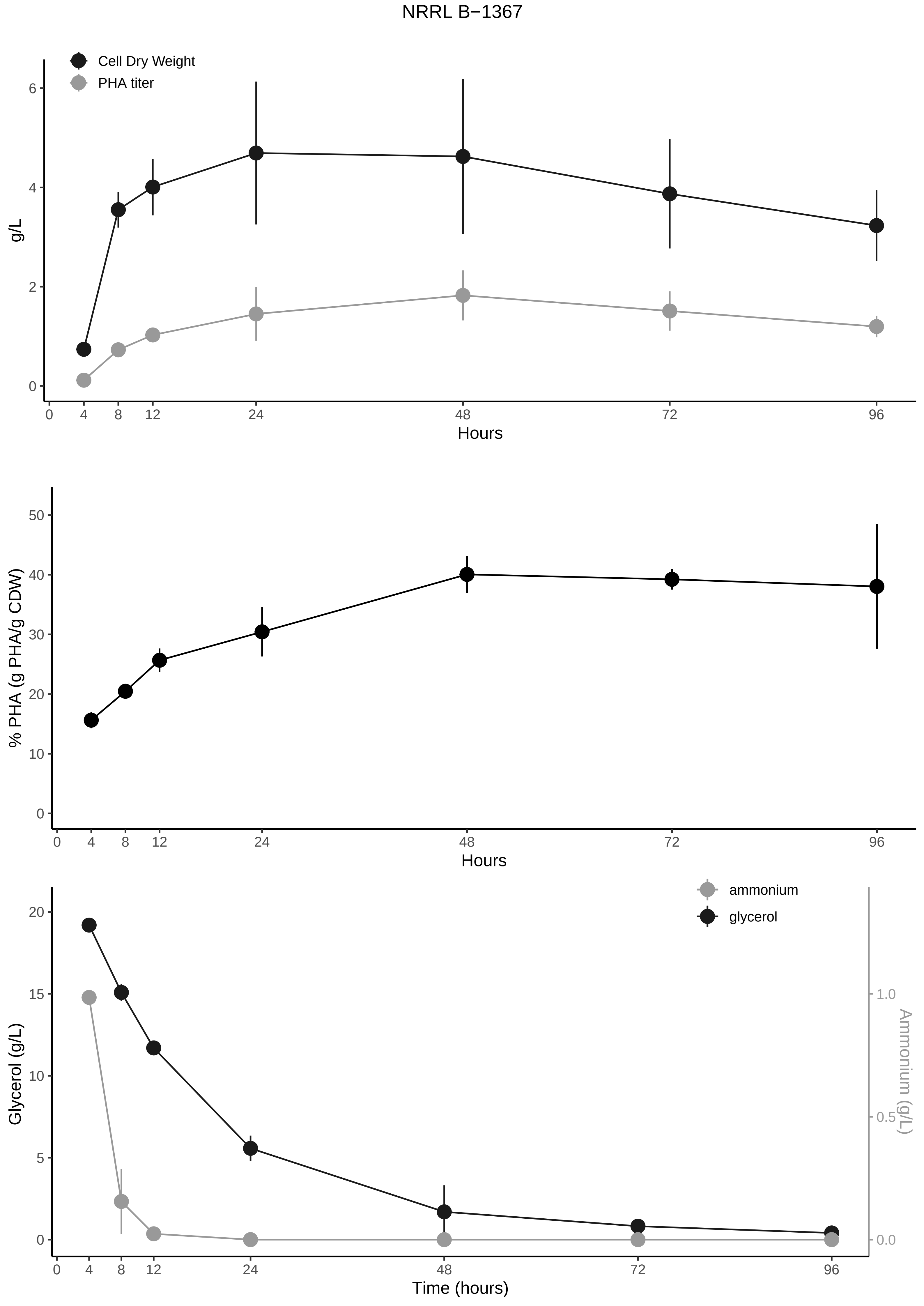
NRRL B−1367
Cell Dry Weight
PHA titer
g/L
Hours
% PHA (g PHA/g CDW)
Hours
ammonium
glycerol
Glycerol (g/L)
Ammonium (g/L)
Time (hours)

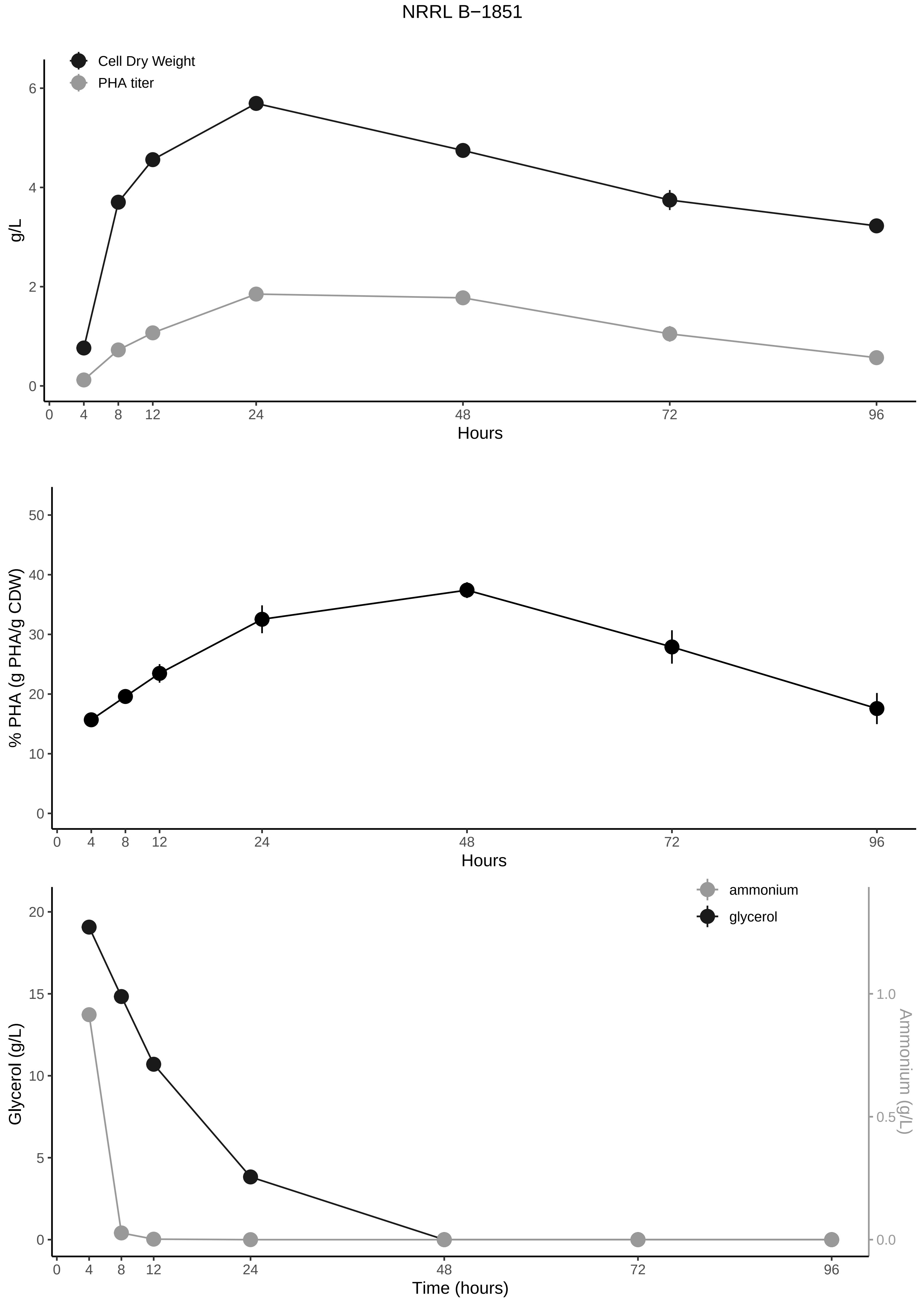

NRRL B−1851
Cell Dry Weight
PHA titer
g/L
Hours
% PHA (g PHA/g CDW)
Hours
ammonium
glycerol
Glycerol (g/L)
Ammonium (g/L)
Time (hours)

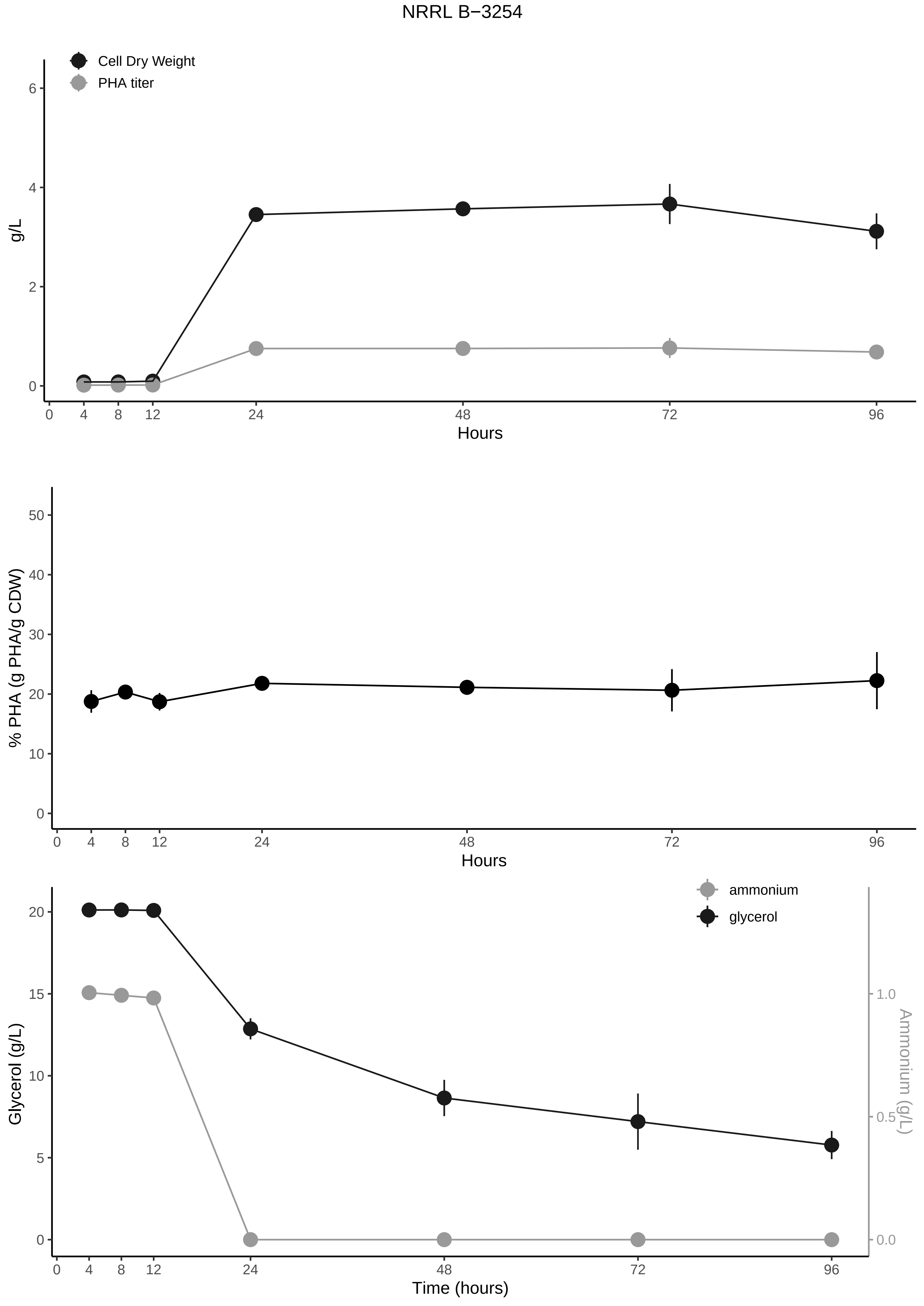

NRRL B−3254
Cell Dry Weight
PHA titer
g/L
0
2
4
6
0
4
8
12
24
48
72
96
Hours
% PHA (g PHA/g CDW)
0
10
20
30
40
50
0
4
8
12
24
48
72
96
Hours
ammonium
glycerol
Glycerol (g/L)
0
5
10
15
20
Ammonium (g/L)
0.0
0.5
1.0
0
4
8
12
24
48
72
96
Time (hours)

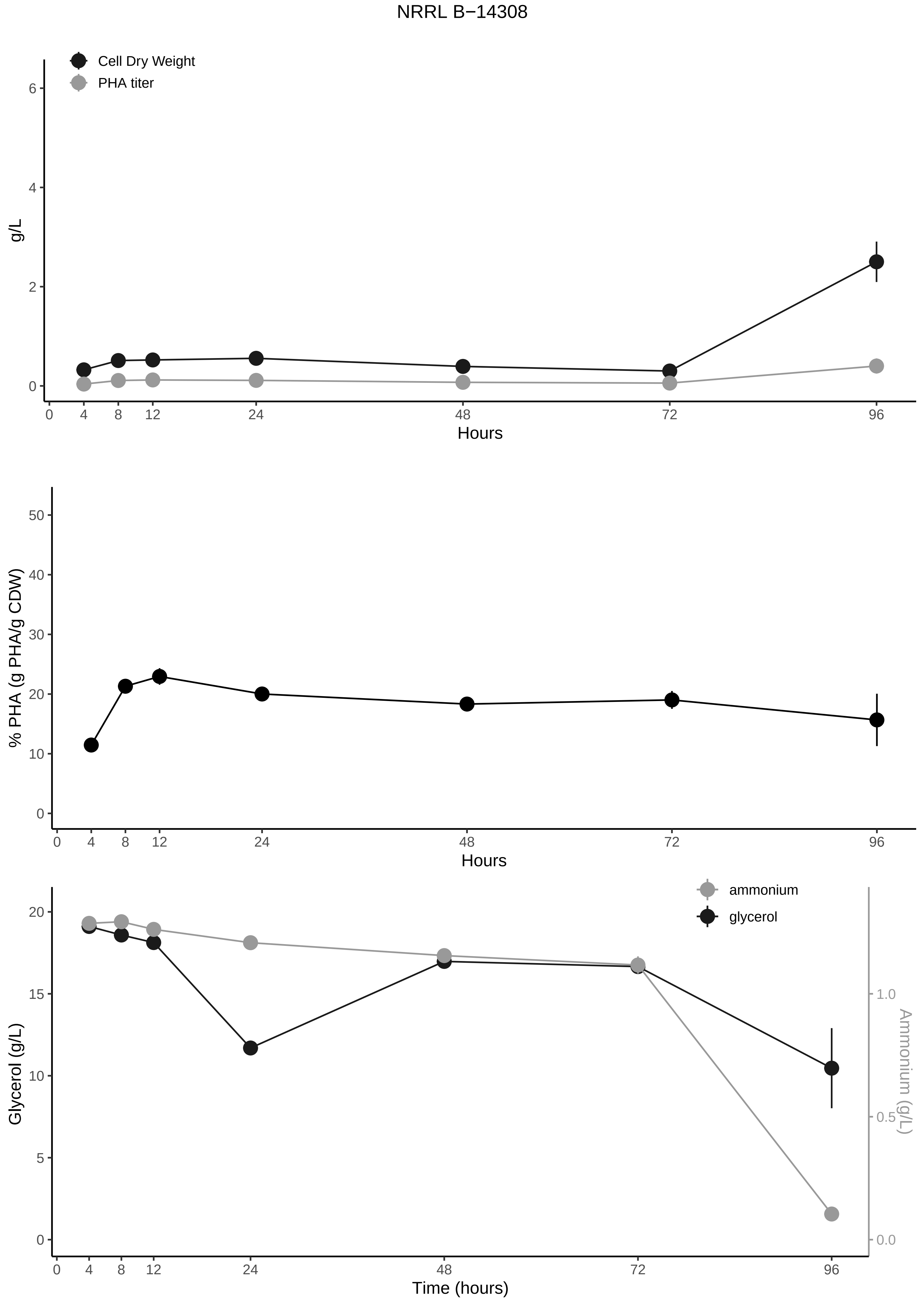

NRRL B−14308
Cell Dry Weight
PHA titer
g/L
Hours
% PHA (g PHA/g CDW)
Hours
ammonium
glycerol
Glycerol (g/L)
Ammonium (g/L)
Time (hours)

Sample key: x, y, z correspond to replicates. Suffix corresponds to timepoint (h).

Sample	CDW (g/L)	PHA (g/L)	%PHA	residual glycerol (g/L)	residual NH4SO4 (g/L)	yield (mg/g)	productivity (g/L/h)
NRS 269-x-4	0.35	0.024	6.7	19.21	0.50	29.6	0.006
NRS 269-y-4	0.32	0.024	7.6	19.19	0.50	30.1	0.006
NRS 269-z-4	0.34	0.023	6.6	19.17	0.51	27.2	0.006
NRS 269-x-8	2.76	0.563	20.4	15.36	0.24	121.5	0.070
NRS 269-y-8	2.71	0.547	20.1	15.45	0.27	120.3	0.068
NRS 269-z-8	2.84	0.564	19.9	15.22	0.19	117.9	0.070
NRS 269-x-12	3.60	0.944	26.2	10.36	0.01	98.0	0.079
NRS 269-y-12	4.25	0.986	23.2	10.77	0.01	106.9	0.082
NRS 269-z-12	3.82	1.005	26.3	10.76	0.01	108.9	0.084
NRS 269-x-24	5.85	2.065	35.3	3.09	-0.01	122.1	0.086
NRS 269-y-24	6.27	1.988	31.7	3.67	-0.02	121.7	0.083
NRS 269-z-24	3.76	1.389	37.0	6.62	-0.02	103.8	0.058
NRS 269-x-48	5.61	2.276	40.6	0.00	-0.02	113.8	0.047
NRS 269-y-48	6.03	2.305	38.2	0.00	-0.02	115.2	0.048
NRS 269-z-48	4.05	1.646	40.6	5.69	-0.02	115.0	0.034
NRS 269-x-72	4.94	1.734	35.1	0.23	-0.02	87.7	0.024
NRS 269-y-72	4.55	1.558	34.2	0.22	-0.02	78.7	0.022
NRS 269-z-72	3.31	1.626	49.1	3.59	-0.02	99.1	0.023
NRS 269-x-96	4.44	1.592	35.8	0.22	-0.02	80.5	0.017
NRS 269-y-96	2.70	0.846	31.3	0.23	-0.01	42.8	0.009
NRS 269-z-96	2.24	1.167	52.1	1.76	-0.01	64.0	0.012
YYBm1-x-4	0.80	0.042	5.2	19.70	0.90	137.7	0.010
YYBm1-y-4	0.75	0.039	5.2	19.40	0.90	64.3	0.010
YYBm1-z-4	0.75	0.038	5.0	19.74	0.88	143.1	0.009
YYBm1-x-8	3.12	1.313	42.1	15.81	0.01	313.1	0.164
YYBm1-y-8	2.79	1.154	41.5	15.97	0.10	286.3	0.144
YYBm1-z-8	2.94	1.245	42.3	15.97	0.03	309.2	0.156
YYBm1-x-12	3.30	0.144	4.4	13.39	0.00	21.7	0.012
YYBm1-y-12	2.82	0.114	4.0	14.32	0.00	20.0	0.009
YYBm1-z-12	3.01	0.145	4.8	13.76	0.00	23.3	0.012
YYBm1-x-24	3.70	0.128	3.5	9.32	-0.03	12.0	0.005
YYBm1-y-24	2.79	0.092	3.3	11.65	-0.01	11.0	0.004
YYBm1-z-24	3.53	0.122	3.4	9.56	-0.03	11.7	0.005
YYBm1-x-48	3.11	0.062	2.0	6.16	-0.03	4.4	0.001
YYBm1-y-48	2.98	0.061	2.1	9.33	-0.03	5.8	0.001
YYBm1-z-48	2.98	0.061	2.0	6.25	-0.03	4.4	0.001
YYBm1-x-72	2.50	0.067	2.7	3.52	-0.03	4.1	0.001
YYBm1-y-72	2.42	0.048	2.0	7.64	-0.03	3.9	0.001

YYBm1-z-72	2.38	0.063	2.6	3.91	-0.03	3.9	0.001
YYBm1-x-96	2.13	0.056	2.6	1.35	-0.03	3.0	0.001
YYBm1-y-96	2.21	0.039	1.7	6.67	-0.03	2.9	0.000
YYBm1-z-96	1.80	0.048	2.7	2.05	-0.03	2.7	0.001
DSM 319-x-4	0.47	0.025	5.3	19.98	1.03	1242.4	0.006
DSM 319-y-4	0.48	0.028	5.7	20.06	1.02	-437.2	0.007
DSM 319-z-4	0.48	0.028	5.9	19.95	1.01	597.9	0.007
DSM 319-x-8	2.58	0.723	28.0	16.28	0.25	194.3	0.090
DSM 319-y-8	2.59	0.740	28.5	16.14	0.22	191.9	0.092
DSM 319-z-8	3.00	0.780	26.0	15.32	0.01	166.6	0.098
DSM 319-x-12	2.72	0.783	28.8	14.26	0.07	136.4	0.065
DSM 319-y-12	2.89	0.647	22.4	13.73	0.00	103.3	0.054
DSM 319-z-12	3.48	0.871	25.0	11.87	0.00	107.2	0.073
DSM 319-x-24	3.42	0.098	2.9	8.11	-0.03	8.2	0.004
DSM 319-y-24	3.40	0.126	3.7	7.03	-0.03	9.7	0.005
DSM 319-z-24	4.53	0.085	1.9	6.53	-0.03	6.3	0.004
DSM 319-x-48	3.55	0.082	2.3	2.64	-0.03	4.7	0.002
DSM 319-y-48	3.40	0.108	3.2	1.50	-0.03	5.8	0.002
DSM 319-z-48	4.21	0.076	1.8	0.81	-0.03	4.0	0.002
DSM 319-x-72	2.82	0.056	2.0	0.00	-0.03	2.8	0.001
DSM 319-y-72	2.86	0.077	2.7	0.00	-0.03	3.8	0.001
DSM 319-z-72	3.00	0.044	1.5	0.00	-0.03	2.2	0.001
DSM 319-x-96	2.69	0.027	1.0	0.00	-0.03	1.3	0.000
DSM 319-y-96	2.56	0.032	1.2	0.00	-0.01	1.6	0.000
DSM 319-z-96	2.68	0.027	1.0	0.00	0.02	1.4	0.000
NRRL B-349-x-4	0.38	0.033	8.8	19.70	0.95	109.9	0.008
NRRL B-349-y-4	0.39	0.033	8.6	19.70	0.98	111.8	0.008
NRRL B-349-z-4	0.39	0.028	7.1	19.95	0.96	603.0	0.007
NRRL B-349-x-8	1.74	0.219	12.5	17.57	0.47	90.0	0.027
NRRL B-349-y-8	1.84	0.297	16.2	17.34	0.41	111.4	0.037
NRRL B-349-z-8	1.67	0.292	17.5	17.74	0.48	128.9	0.036
NRRL B-349-x-12	2.83	0.559	19.8	14.35	0.01	98.9	0.047
NRRL B-349-y-12	2.76	0.500	18.2	14.49	0.02	90.8	0.042
NRRL B-349-z-12	2.98	0.563	18.9	14.34	0.01	99.4	0.047
NRRL B-349-x-24	3.33	0.440	13.2	10.55	0.00	46.5	0.018
NRRL B-349-y-24	3.15	0.487	15.5	10.73	0.00	52.6	0.020
NRRL B-349-z-24	3.38	0.659	19.5	10.45	0.00	69.0	0.027
NRRL B-349-x-48	2.69	0.521	19.4	8.13	-0.01	43.9	0.011
NRRL B-349-y-48	2.58	0.410	15.9	8.51	-0.01	35.7	0.009
NRRL B-349-z-48	2.70	0.617	22.8	7.97	-0.01	51.2	0.013
NRRL B-349-x-72	2.42	0.465	19.2	6.00	-0.01	33.2	0.006

NRRL B-349-y-72	2.36	0.349	14.8	6.60	-0.01	26.1	0.005
NRRL B-349-z-72	2.56	0.440	17.2	5.95	-0.01	31.3	0.006
NRRL B-349-x-96	2.21	0.432	19.5	4.41	-0.01	27.7	0.004
NRRL B-349-y-96	2.13	0.275	12.9	5.22	-0.01	18.6	0.003
NRRL B-349-z-96	2.43	0.392	16.1	4.20	-0.01	24.8	0.004
NRRL B-350-x-4	0.05	0.010	19.9	19.96	0.97	220.6	0.002
NRRL B-350-y-4	0.07	0.008	11.9	20.00	0.99	4793.4	0.002
NRRL B-350-z-4	0.06	0.007	13.3	20.00	0.99	-1525.9	0.002
NRRL B-350-x-8	0.12	0.014	12.0	19.94	0.97	222.0	0.002
NRRL B-350-y-8	0.10	0.012	11.4	19.98	0.98	487.3	0.001
NRRL B-350-z-8	0.12	0.014	11.3	19.95	0.99	268.6	0.002
NRRL B-350-x-12	1.01	0.172	17.0	18.80	0.76	143.2	0.014
NRRL B-350-y-12	0.92	0.147	15.9	18.89	0.80	132.1	0.012
NRRL B-350-z-12	0.92	0.146	15.8	18.88	0.79	129.8	0.012
NRRL B-350-x-24	4.77	1.294	27.1	6.94	0.00	99.1	0.054
NRRL B-350-y-24	5.16	1.319	25.5	7.25	0.00	103.5	0.055
NRRL B-350-z-24	4.21	0.984	23.4	7.89	0.00	81.2	0.041
NRRL B-350-x-48	5.04	1.906	37.8	0.00	0.00	95.3	0.040
NRRL B-350-y-48	5.38	1.860	34.6	0.00	0.00	93.0	0.039
NRRL B-350-z-48	4.94	2.010	40.6	0.87	0.00	105.1	0.042
NRRL B-350-x-72	4.27	1.385	32.4	0.00	0.00	69.2	0.019
NRRL B-350-y-72	3.91	0.835	21.4	0.00	0.00	41.8	0.012
NRRL B-350-z-72	4.27	1.903	44.6	0.00	0.00	95.2	0.026
NRRL B-350-x-96	3.63	0.867	23.9	0.00	-0.01	43.4	0.009
NRRL B-350-y-96	2.98	0.407	13.6	0.00	-0.01	20.3	0.004
NRRL B-350-z-96	3.75	1.246	33.2	0.00	0.00	62.3	0.013
NRRL B-352-x-4	0.07	0.019	25.9	20.35	0.94	-54.8	0.005
NRRL B-352-y-4	0.09	0.022	24.9	20.38	0.97	-57.5	0.005
NRRL B-352-z-4	0.08	0.018	21.3	20.31	1.00	-56.8	0.004
NRRL B-352-x-8	0.12	0.022	18.2	20.49	1.01	-45.1	0.003
NRRL B-352-y-8	0.19	0.024	12.8	20.35	0.98	-67.8	0.003
NRRL B-352-z-8	0.11	0.023	21.1	20.36	0.98	-64.1	0.003
NRRL B-352-x-12	0.15	0.025	16.2	20.26	0.95	-96.1	0.002
NRRL B-352-y-12	0.16	0.023	14.8	20.30	0.98	-77.8	0.002
NRRL B-352-z-12	0.17	0.024	13.6	20.27	1.00	-87.8	0.002
NRRL B-352-x-24	2.68	0.326	12.2	14.41	-0.02	58.4	0.014
NRRL B-352-y-24	2.98	0.429	14.4	13.52	-0.02	66.2	0.018
NRRL B-352-z-24	3.04	0.416	13.7	12.58	-0.02	56.0	0.017
NRRL B-352-x-48	4.31	1.482	34.3	0.36	-0.02	75.5	0.031
NRRL B-352-y-48	4.48	1.578	35.2	0.16	-0.02	79.5	0.033
NRRL B-352-z-48	4.36	1.851	42.5	0.00	-0.02	92.5	0.039

NRRL B-352-x-72	3.34	0.952	28.5	0.00	-0.02	47.6	0.013
NRRL B-352-y-72	3.43	1.012	29.6	0.06	-0.02	50.8	0.014
NRRL B-352-z-72	3.57	1.106	31.0	0.00	-0.02	55.3	0.015
NRRL B-352-x-96	2.92	0.383	13.1	0.06	-0.02	19.2	0.004
NRRL B-352-y-96	2.89	0.403	14.0	0.06	-0.02	20.2	0.004
NRRL B-352-z-96	2.91	0.360	12.4	0.06	0.00	18.0	0.004
NRRL B-1367-x-4	0.74	0.111	15.1	19.33	0.98	166.2	0.028
NRRL B-1367-y-4	0.77	0.112	14.6	19.12	0.98	127.7	0.028
NRRL B-1367-z-4	0.71	0.122	17.2	19.12	1.00	138.7	0.030
NRRL B-1367-x-8	3.43	0.679	19.8	15.27	0.21	143.6	0.085
NRRL B-1367-y-8	3.27	0.678	20.7	15.47	0.26	149.7	0.085
NRRL B-1367-z-8	3.96	0.825	20.9	14.50	0.01	150.1	0.103
NRRL B-1367-x-12	3.58	0.996	27.8	11.91	0.01	123.0	0.083
NRRL B-1367-y-12	3.78	0.903	23.9	11.89	0.05	111.4	0.075
NRRL B-1367-z-12	4.66	1.181	25.3	11.30	0.01	135.6	0.098
NRRL B-1367-x-24	3.29	0.857	26.0	6.46	-0.04	63.3	0.036
NRRL B-1367-y-24	4.62	1.582	34.2	5.01	-0.04	105.6	0.066
NRRL B-1367-z-24	6.17	1.911	31.0	5.25	-0.04	129.6	0.080
NRRL B-1367-x-48	2.99	1.257	42.0	3.53	-0.04	76.3	0.026
NRRL B-1367-y-48	4.78	1.992	41.7	0.44	-0.04	101.8	0.042
NRRL B-1367-z-48	6.10	2.224	36.5	1.13	-0.04	117.8	0.046
NRRL B-1367-x-72	2.80	1.152	41.1	2.29	-0.04	65.1	0.016
NRRL B-1367-y-72	3.81	1.441	37.8	0.17	-0.04	72.7	0.020
NRRL B-1367-z-72	5.00	1.937	38.7	0.00	-0.04	96.8	0.027
NRRL B-1367-x-96	2.66	1.331	50.1	1.23	-0.04	70.9	0.014
NRRL B-1367-y-96	3.00	0.947	31.5	0.00	-0.04	47.4	0.010
NRRL B-1367-z-96	4.03	1.310	32.5	0.00	-0.04	65.5	0.014
NRRL B-1851-x-4	0.76	0.123	16.2	19.10	0.91	136.5	0.031
NRRL B-1851-y-4	0.79	0.120	15.2	19.00	0.90	120.1	0.030
NRRL B-1851-z-4	0.74	0.117	15.7	19.10	0.94	130.1	0.029
NRRL B-1851-x-8	3.72	0.715	19.2	14.54	0.00	131.0	0.089
NRRL B-1851-y-8	3.72	0.711	19.1	14.81	0.00	137.0	0.089
NRRL B-1851-z-8	3.67	0.750	20.4	15.15	0.08	154.6	0.094
NRRL B-1851-x-12	4.64	1.064	22.9	10.52	0.00	112.2	0.089
NRRL B-1851-y-12	4.56	1.015	22.2	10.68	0.00	108.9	0.085
NRRL B-1851-z-12	4.48	1.130	25.2	10.90	0.00	124.1	0.094
NRRL B-1851-x-24	5.63	1.954	34.7	4.03	-0.04	122.3	0.081
NRRL B-1851-y-24	5.80	1.745	30.1	3.62	-0.04	106.6	0.073
NRRL B-1851-z-24	5.65	1.853	32.8	3.82	-0.04	114.6	0.077
NRRL B-1851-x-48	4.59	1.748	38.1	0.00	-0.04	87.4	0.036
NRRL B-1851-y-48	4.85	1.858	38.3	0.00	-0.04	92.9	0.039

NRRL B-1851-z-48	4.79	1.719	35.9	0.00	-0.04	86.0	0.036
NRRL B-1851-x-72	3.51	0.867	24.7	0.00	-0.04	43.3	0.012
NRRL B-1851-y-72	3.87	1.147	29.7	0.00	-0.04	57.4	0.016
NRRL B-1851-z-72	3.86	1.132	29.4	0.00	-0.04	56.6	0.016
NRRL B-1851-x-96	3.07	0.451	14.7	0.00	-0.04	22.6	0.005
NRRL B-1851-y-96	3.31	0.604	18.3	0.00	-0.04	30.2	0.006
NRRL B-1851-z-96	3.29	0.651	19.8	0.00	-0.04	32.5	0.007
NRRL B-3254-x-4	0.08	0.016	20.7	19.97	1.00	551.1	0.004
NRRL B-3254-y-4	0.10	0.016	16.9	20.17	1.01	-96.4	0.004
NRRL B-3254-z-4	0.07	0.013	18.7	20.20	1.01	-63.0	0.003
NRRL B-3254-x-8	0.09	0.018	19.5	20.01	0.99	-2946.7	0.002
NRRL B-3254-y-8	0.08	0.016	19.8	20.16	1.00	-98.4	0.002
NRRL B-3254-z-8	0.07	0.015	21.7	20.18	1.00	-83.6	0.002
NRRL B-3254-x-12	0.10	0.021	20.4	19.97	0.98	766.7	0.002
NRRL B-3254-y-12	0.11	0.019	17.7	20.12	0.98	-149.2	0.002
NRRL B-3254-z-12	0.08	0.015	18.0	20.16	0.99	-90.6	0.001
NRRL B-3254-x-24	3.47	0.771	22.2	12.20	-0.03	98.9	0.032
NRRL B-3254-y-24	3.38	0.699	20.7	13.49	-0.03	107.3	0.029
NRRL B-3254-z-24	3.51	0.789	22.5	12.90	-0.03	111.1	0.033
NRRL B-3254-x-48	3.57	0.770	21.5	7.60	-0.03	62.1	0.016
NRRL B-3254-y-48	3.55	0.700	19.7	9.80	0.00	68.6	0.015
NRRL B-3254-z-48	3.58	0.792	22.1	8.53	-0.03	69.1	0.016
NRRL B-3254-x-72	3.93	0.953	24.3	6.30	-0.04	69.5	0.013
NRRL B-3254-y-72	3.87	0.792	20.5	9.18	-0.02	73.2	0.011
NRRL B-3254-z-72	3.20	0.550	17.2	6.13	-0.04	39.6	0.008
NRRL B-3254-x-96	3.26	0.752	23.1	5.06	-0.04	50.3	0.008
NRRL B-3254-y-96	3.38	0.579	17.1	6.73	-0.04	43.6	0.006
NRRL B-3254-z-96	2.70	0.719	26.6	5.52	-0.04	49.6	0.007
NRRL B-14308-x-4	0.29	0.034	11.5	19.03	1.28	34.6	0.008
NRRL B-14308-y-4	0.39	0.042	10.8	19.29	1.29	58.7	0.010
NRRL B-14308-z-4	0.29	0.035	12.1	19.01	1.29	35.5	0.009
NRRL B-14308-x-8	0.51	0.105	20.4	18.58	1.29	73.8	0.013
NRRL B-14308-y-8	0.53	0.114	21.6	18.60	1.30	81.2	0.014
NRRL B-14308-z-8	0.49	0.108	22.0	18.58	1.29	76.2	0.014
NRRL B-14308-x-12	0.51	0.116	22.8	18.21	1.25	65.0	0.010
NRRL B-14308-y-12	0.52	0.113	21.6	18.09	1.27	59.5	0.009
NRRL B-14308-z-12	0.53	0.130	24.4	18.08	1.26	67.7	0.011
NRRL B-14308-x-24	0.55	0.112	20.2	0.00	1.20	5.6	0.005
NRRL B-14308-y-24	0.55	0.104	19.0	17.53	1.21	42.2	0.004
NRRL B-14308-z-24	0.56	0.118	20.9	17.55	1.21	48.1	0.005
NRRL B-14308-x-48	0.47	0.089	19.0	16.75	1.15	27.2	0.002

NRRL B-14308-y-48	0.34	0.060	17.8	17.17	1.14	21.3	0.001
NRRL B-14308-z-48	0.37	0.068	18.2	17.01	1.17	22.7	0.001
NRRL B-14308-x-72	0.28	0.057	20.3	16.64	1.13	16.9	0.001
NRRL B-14308-y-72	0.35	0.061	17.4	16.42	1.08	17.1	0.001
NRRL B-14308-z-72	0.27	0.052	19.4	16.92	1.15	16.9	0.001
NRRL B-14308-x-96	2.07	0.225	10.8	13.08	0.25	32.4	0.002
NRRL B-14308-y-96	2.54	0.493	19.4	8.24	0.03	41.9	0.005
NRRL B-14308-z-96	2.89	0.485	16.8	10.07	0.03	48.9	0.005
